# The Role of Glia in Alpha-Synucleinopathies

**DOI:** 10.1007/s12035-012-8340-3

**Published:** 2012-09-02

**Authors:** Lisa Fellner, Nadia Stefanova

**Affiliations:** Division of Neurobiology, Department of Neurology, Innsbruck Medical University, Anichstrasse 35, 6020 Innsbruck, Austria

**Keywords:** α-Synuclein, Astroglia, Microglia, Multiple system atrophy, Oligodendroglia, Parkinson’s disease

## Abstract

α-Synuclein (AS)-positive inclusions are the pathological hallmark of Parkinson’s disease (PD), dementia with Lewy bodies (DLB) and multiple system atrophy (MSA), all belonging to the category of α-synucleinopathies. α-Synucleinopathies represent progressive neurodegenerative disorders characterised by increasing incidences in the population over the age of 65. The relevance of glial reactivity and dysfunction in α-synucleinopathies is highlighted by numerous experimental evidences. Glial AS inclusion pathology is prominent in oligodendroglia of MSA (glial cytoplasmic inclusions) and is a common finding in astroglial cells of PD and DLB, resulting in specific dysfunctional responses. Involvement of AS-dependent astroglial and microglial activation in neurodegenerative mechanisms, and therefore in disease initiation and progression, has been suggested. The aim of this review is to summarise and discuss the multifaceted responses of glial cells in α-synucleinopathies. The beneficial, as well as detrimental, effects of glial cells on neuronal viability are taken into consideration to draw an integrated picture of glial roles in α-synucleinopathies. Furthermore, an overview on therapeutic approaches outlines the difficulties of translating promising experimental studies into successful clinical trials targeting candidate glial pathomechanisms.

## General Background

α-Synuclein (AS) belongs to a distinct protein family including α-, β- and γ-synuclein. It is natively unfolded and consists of 140 amino acids. Its importance in synaptic structure and presynaptic terminal size was recently demonstrated in αβγ-knockout mice [1]. Furthermore, AS plays an important role in the mechanisms of folding and re-folding of synaptic proteins, acting in close connection with cysteine string protein-α and SNARE proteins [2].

The term α-synucleinopathies comprises progressive, neurodegenerative diseases including Parkinson’s disease (PD), dementia with Lewy bodies (DLB) and multiple system atrophy (MSA) with the major pathological hallmark of AS-positive inclusions in neuronal and glial cells. Neuronal inclusions, Lewy bodies (LBs) and Lewy neurites (LNs) are characteristic for PD and DLB, while AS-positive glial cytoplasmic inclusions (GCIs) are distinctive in MSA and occur predominantly in oligodendroglial cells [3,4]. Astroglial AS-positive inclusions may also occur in PD [5,6]. PD pathology has been partly related to point mutations [7,8] or duplications [9] and triplications [10,11] of the SNCA gene. Moreover, SNCA variants can increase the risk of developing PD and MSA [12,13]. AS inclusion formation may be related to posttranslational modifications of AS (nitration, ubiquitination and phosphorylation) which can lead to pathological accumulation of AS and enhance the progression of α-synucleinopathies [14–16]. Involvement of impaired AS clearance through autophagy pathways is also suggested to be involved in the generation of AS inclusions in PD and DLB [17,18]. A correlation between the aggregation of AS and neuronal cell loss and disease progression respectively was demonstrated in MSA [19] and also suggested in PD/DLB according to Braak staging [20]. Moreover, prion-like cell-to-cell propagation of AS has been proposed recently as a major contributor to disease progression in α-synucleinopathies [21–23].

Since the first description of glial cells (glia meaning glue) by Rudolf Virchow in 1864, the view of glial cells as mere substrate for neurons has changed by evidence, indicating the role of glial cells in the support of neuronal survival, synaptic function and local immunity [24,25]. Furthermore, the importance of glial cells is now extended towards a crucial role in the initiation and progression of different diseases of the CNS, including α-synucleinopathies [26–29]. Glial dysfunction in α-synucleinopathies not only comprises the above-mentioned AS-positive inclusion pathology in glia but also an over-activated state of microglial and astroglial cells, termed reactive microgliosis and astrogliosis. On different stimuli, e.g. injury or infection, microglial and astroglial cells get activated [30,31]. Activation is associated with morphological changes, release of trophic and inflammatory factors and, in regard to microglia, also clearance of dead or damaged cells [30–34]. These changes can be crucial for neuronal survival [32–34]. However, regarding chronic disease conditions of the CNS like neurodegenerative diseases, astroglia and microglia can get over activated. Reactive microgliosis and astrogliosis can lead to neurotoxicity and increased tissue damage after the release of (pro-)inflammatory cytokines, reactive oxygen species (ROS) and nitric oxide (NO) [35–40]. Alternatively, oligodendroglial cells show an increased vulnerability to oxidative stress and cytokines, resulting in demyelination, diminished trophic support, cellular dysfunction and cell death which affect neuronal survival [41,42].

## Neuronal α-Synucleinopathies

α-Synucleinopathies show frequent incidences among the population over the age of 65. PD affects about 3 % of the general population over the age of 65 and, therefore, is the most common neurodegenerative movement disorder [43]. Furthermore, it is characterised by relentless disease progression [44]. DLB has a frequency of 20 % regarding all cases of dementia analysed by autopsy [45]. PD and DLB show various degrees of neurodegeneration of dopaminergic neurons in substantia nigra pars compacta (SNpc) and dopaminergic terminals in the striatum, as well as degeneration of extra-nigral structures including noradrenergic system, cholinergic system, serotonergic system, limbic structures and cerebral cortex [46–48]. The aggregation of AS in neuronal cells is the major pathological hallmark of PD and DLB, including LBs and LNs [4,49]. PD also features abnormal aggregations of AS in astroglial cells [5,6]. LB pathology is mostly present at the sites of neuronal loss [48]. However, evidence shows that the occurrence of LBs in the SNpc must not necessarily lead to neuronal death and a high number of neurons undergo apoptosis without the occurrence of AS aggregates [50,51]. This leads to the assumption that other factors may have a major influence on neuronal degeneration and, therefore, on the progression of these diseases. Hence, the role of microglial [28,52,53] and astroglial [5] activation in PD and DLB progression comes into consideration. However, the role of both cell types, microglia and astroglia, is still unclear in PD and DLB because of their controversial beneficial and toxic effects on neurons [54,55]. Furthermore, AS-positive inclusions were shown in oligodendroglial cells of PD brains [6]; however, in contrast to MSA, oligodendroglial cells seem to play an inferior role in the initiation of PD and DLB but may have a possible involvement in the late stages of disease progression [29].

Astroglial cells have been shown to get activated in PD and DLB. Different reports exist on astroglial activation, claiming no or mild astrogliosis [56,57] in contrast to massive astrogliosis [58] in post-mortem PD brains. Furthermore, astroglial cells show AS-positive inclusion pathology [6], which may lead to a different reactivity pattern in PD and DLB [5,19]. AS overexpression in murine astroglial cells leads to neuroinflammation and microglial activation, and in consequence to oxidative stress [59], providing a major link of AS astroglial pathology with neuroinflammation/microgliosis and oxidative stress that may also promote neurodegeneration. A possible explanation for the AS-positive inclusions in astroglial cells in PD brains was provided by Lee and colleagues [23]. They show that astroglial cells can endocytose AS released from neurons and form inclusions similar to LBs in a time-dependent manner. Importantly, the transfer of AS from neurons to astroglia leads to increased production of tumour necrosis factor α (TNF-α) and chemokine ligand 1 by astroglial cells, and results in enhanced neurodegeneration [23].

An upregulation of interferon-γ (IFN-γ) receptor on astroglia in PD post-mortem brains suggested a neurotoxic reaction after INF-γ activation [54,60]. Furthermore, astroglial cells in the ventral midbrain of PD brains show an enhanced expression of myeloperoxidase (MPO), a key enzyme related to oxidative stress during inflammation [61]. However, astroglial cells also seem to function in a contrary direction by the production of anti-oxidative and anti-inflammatory agents. A beneficial function of astroglial cells in PD and DLB seems to be the release of neurotrophic factors, e.g. brain-derived neurotrophic factor [55]. Moreover, the activity of glutathione peroxidase (GPx), a crucial protective enzyme against oxidative damage, has been associated with astrocytic proliferation and showed an enhancement of 30 % in the substantia nigra (SN) of PD brains [62]. Enhanced levels of glial fibrillary acidic protein were associated with increased GPx activity in PD brains [63], suggesting a fundamental role of astroglia in neuronal protection against oxidative stress.

Experimental models of PD reveal further the involvement of astrocytes not only in neurotoxicity but also in neuronal protection. Astroglial cells in Parkinsonian monkeys, intoxicated with 1-methyl-4-phenyl-1,2,3,6-tetrahydropyridine (MPTP), show an upregulated expression of the IFN-γ receptor similar to human post-mortem brains. Moreover, TNF-α immunoreactivity was observed almost exclusively in astroglial cells, associated with an increased number of astrocytes even years after the MPTP intoxication, suggesting a role in neurodegeneration [64]. Selective astroglial expression of mutant A53T AS in an inducible mouse model led to rapidly progressive paralysis most likely caused by widespread astrogliosis, degeneration of spinal cord motor and dopaminergic neurons [59]. An alternative pathogenic pathway of astroglia-mediated neurotoxicity could be related to morphological and functional alterations in astroglial mitochondria and a disturbed secretion of factors crucial for neuronal differentiation as demonstrated in a genetic mouse model overexpressing mutant AS [65]. In a recent study, cerebrospinal fluid of PD patients was added to an astroglial cell culture, and a decrease in proliferation rate as well as increased contents of AS on day 7 was observed [66]. Upregulated expression of interleukine-6 (IL-6) by astroglial cells upon AS treatment has been shown in vitro, supporting the evidence of astroglia-triggered neuroinflammatory response [67]. However, astroglial release of glial cell line-derived neurotrophic factor (GDNF) may favour neuronal protection in SNpc [68]. Glutathione, another agent reported to have an antioxidant character with beneficial functions in PD, was demonstrated to be released by astroglial cells activated by 6-OHDA-injured dopaminergic neurons [69]. Hydrogen sulphide, a potential anti-inflammatory and neuroprotective agent produced by astroglial cells, was found downregulated upon inflammatory activation of astroglia or microglia, suggesting a possible mechanism relevant to PD pathogenesis [70].

**Fig. 1:**
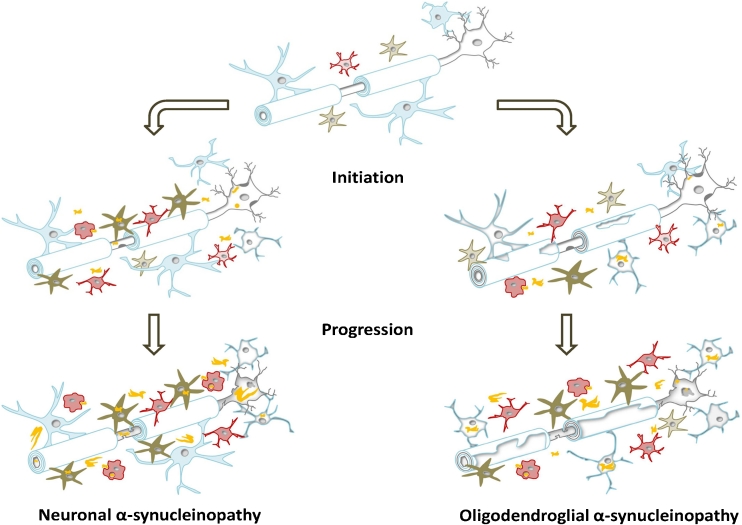
Characteristic cellular changes in the CNS during disease initiation and progression in neuronal (PD/DLB) and oligodendroglial (MSA) α-synucleinopathies. In the healthy brain, microglial cells (*red*) are present in a quiescent or resting state. Their main task is to scan the environment for injury or infection. Astroglial cells (*green*) are involved in synaptic transmission support, nutrient support and control of extracellular homeostasis, thereby crucial for neuronal viability. Neurotrophic support is also provided by microglial, astroglial and oligodendroglial (myelinating and non-myelinating) cells. Myelinating oligodendroglia (*blue*) are essentially involved in maintaining the myelin sheet and trophic support of myelinated neurites. *Neuronal α-synucleinopathies*: Early in disease, AS aggregations (*yellow*) in neurons (*grey*) and astroglial cells occur, leading to a decreased neuronal viability. Moreover, astroglial cells get activated, resulting in an enhanced release of neurotoxic pro-inflammatory factors. The recruitment of microglial cells starts even before neuronal cell loss occurs, and their arrival at the site of AS accumulation facilitates the production of pro-inflammatory cytokines, as well as oxidative stress. However, beneficial phagocytic microglial activity may be involved in the early clearance of extracellular AS. Later in disease progression, full-blown neuronal inclusion pathology develops, including the formation of LBs and LNs. The build-up of AS in astroglia leads to dysfunctionality and increased neurotoxic activity. The phagocytic microglia appears inefficient to clear extracellular AS and to stop disease progression. Accumulation of AS may even occur in non-myelinating oligodendroglial cells late in disease. All these dramatic changes in the CNS lead to chronic overactivation of glial cells and an enhanced neuronal cell loss. *Oligodendroglial α-synucleinopathies*: At the beginning of MSA, oligodendroglial cells start to accumulate AS in the cytoplasm (origin is still unresolved). Demyelination, oligodendroglial and neuronal degeneration are initiated. Again, activated microglia and astroglia are attracted to the sites of GCI accumulation, and through the release of pro-inflammatory cytokines and oxidative stress, promote the disease. However, microglial phagocytic activity may provide an effort to reduce extracellular AS levels in the CNS. Final stage MSA is presented by AS-positive GCIs, massive oligodendroglial dysfunction (demyelination and disturbed trophic support) and prominent reactive gliosis. Moreover, accumulation of AS in the cytoplasm and nucleus of neurons is frequent. In consequence of all these cellular changes, secondary axonal degeneration and neuronal cell death occur

In summary, it is speculated that astroglia may play a dominant role at least in the initiation of PD related to astroglial AS inclusion pathology [29]. Later on, astroglia may mediate the progression, releasing inflammatory agents and recruiting microglial cells (see Fig. 1). On the other hand, astroglia-mediated secretion of trophic and antioxidant factors should also be taken into account, even though there are insufficient data regarding AS-dependent astroglial neuroprotection. Considering the neuroprotective features of astroglial cells in oxidative stress situations, possible therapeutic options to regulate the astroglial response to AS and chronic disease conditions may be of major interest.

Microglial cells have been shown to be activated in all α-synucleinopathies. In PD, different studies report an accumulation of reactive microglia around AS-positive LBs using in vivo imaging techniques [28] or post-mortem brain analysis [53,71]. In DLB as well, a correlation between microglial activation and LBs in different brain regions has been demonstrated [52]. Microglial activation is shown in different brain regions of PD end-stage cases, including SN, putamen, hippocampus, transentorhinal, cingulated and temporal cortex [72], in all areas of the limbic system, in particular, the dentate gyrus and the CA2/3 region of the hippocampus [73]. To clarify if there is an involvement of microgliosis in PD initiation, Ouchi and colleagues [74] used [^11^C](R)-PK11195 PET scans to image microglial activation in early-stage drug-naïve PD patients [74]. They demonstrated that enhanced microglial activation in midbrain correlates with the loss of dopaminergic terminals in PD. The re-scan of some patients in a follow-up study presented a more prevalent distribution of microgliosis, also affecting extra-striatal regions of the brain [74].

The observations in PD/DLB patients support the recently suggested hypothesis that microglial activation, stimulated by extracellular AS or astroglia, occurs before neurodegeneration in SNpc and is therefore a major participant in the initiation of PD and DLB [29,75]. Furthermore, microglia are suggested to be crucial in the ongoing progression of PD and DLB including, e.g. the secretion of different pro-inflammatory agents [75–78]. Therefore, different experimental models characterise microglial activation by AS and modified forms of AS. The overexpression of wild-type AS in mice presented early microglial activation [78]. The neuronal overexpression of mutant AS forms (A53T and A30P homozygous double mutants) may even enhance microgliosis [75]. In a rat PD model with rAAV-based overexpression of AS in the midbrain, the cell number of microglia increased with the level of AS expression [79]. In addition, dopaminergic cell death influenced the AS-induced microglial activation: Occurrence of dopaminergic cell death lead to a delayed and long-lasting microglial activation, whereas the absence of dopaminergic cell death induced an early and transient activation. Furthermore, the activation profile upon AS overexpression was related to four different types of microglial activation associated with different stages of progression of the neurodegenerative process [79]. In a PD-like mouse model with rAAV-based overexpression of human AS, microglia and the adaptive immune system were activated due to AS alone [80]. AS led to NF-κB/p65 expression, release of pro-inflammatory cytokines and neurodegeneration triggered by microglial cells. The microglial activation was attenuated by the lack of the Fc gamma receptor, suggesting an important role of the adaptive immune system in AS-mediated microglial activation and neurodegeneration [81].

Cell culture models demonstrated that microglial-conditioned release of pro-inflammatory cytokines upon AS treatment was dose-dependent [76,78]. Moreover, microglia treated with mutant (A30P, E46K and A53T) AS resulted in an enhanced microglial activation with increased release of cytokines (IL-6 and IL-10) and chemokines (RANTES and MCP-1) compared to wild-type AS treatment [77]. Wild-type and mutant AS released by neurons led to an enhanced pro-inflammatory response of the mouse microglia cell line BV2 [82], and mutant AS-overexpressing BV2 cells showed an increased release of inflammatory cytokines (e.g. TNF-α, IL-6) [83]. Oxidative stress is another neurotoxic event that occurs after AS-induced microglial activation and may play a crucial role in PD and DLB disease progression. The activation of NADPH oxidase and the production of ROS by AS stimulated microglia led to dopaminergic neuronal loss [84]. Furthermore, mutant AS-overexpressing BV2 cells produced an increased amount of NO [83]. Aggregated and nitrated forms of AS not only led to inflammatory events and oxidative stress but also to enhanced neuronal cell death, as shown in mesencephalic neuron–microglia co-cultures [84,85].

However, activated microglial cells are not only involved in the neuroinflammation and neurodegeneration processes in PD and DLB, they may also play a fundamental role in the clearance of damaged or dead cells and AS [32,84,86], thereby supporting neuronal survival. Microglial cells show an enhanced phagocytic activity when treated with monomeric AS; on the contrary, aggregated AS inhibits microglial phagocytosis in vitro [86]. Microglial cells are capable of fast AS degradation with an intracellular AS half-life of about 4 h, compared to astroglial and neuronal degradation comprising less than half of the degradation time [87]. Regarding microglial phagocytosis of AS, Toll-like receptors (TLRs) seem to play an important part in recognition and internalisation of AS. The pattern-recognition receptors TLR2 and TLR4 were identified as important players in microglial activation [88,89]. Recently, it was demonstrated that TLR4 ablation leads to a disturbed clearance of AS by microglia [89].

To summarise, microglia plays a fundamental but dual role in neuronal α-synucleinopathies. On the one hand, microglial cells recognise extracellular AS released by damaged neurons; moreover, they are crucial in the clearance of this protein [86,87]. On the other hand, AS may trigger microglial overactivation and, further, this can lead to the production of pro-inflammatory agents and to oxidative stress and as a consequence to enhanced neurodegeneration and neuronal cell death [84].

The role of oligodendroglial pathology in PD and DLB does not seem to be a leading one. Occasional oligodendroglial AS-positive inclusions have been reported in clinically overt PD cases [6,90]. Moreover, complement-activated oligodendroglial cells were found in some brain regions of PD and DLB cases [91,92]. Oligodendrocytes show a higher susceptibility in PD and DLB regarding the lack or decrease of myelinated axons of AS-affected neurons [93,94]. These data indicate that oligodendroglial cells could be involved in the late disease progression of PD and DLB. However, an involvement of oligodendroglial pathology in disease initiation and/or progression in neuronal α-synucleinopathies lacks sufficient evidence at present [29].

## Oligodendroglial α-Synucleinopathy

MSA is a progressive neurodegenerative disease of unknown aetiology with a prevalence of about 4.4 new cases per 100,000 year^−1^ [95], and the mean age at onset of first symptoms is 52–57 years [96,97]. The term MSA was first used by Graham and Oppenheimer in 1969 to merge the variable diagnoses of striatonigral degeneration, olivopontocerebellar ataxia, Shy–Drager syndrome and orthostatic hypotension [98,99]. Now, two major clinical subtypes are defined: (1) the MSA-C subtype presents with predominant cerebellar ataxia and (2) the MSA-P subtype shows prevalent Parkinsonism [100]. Progressive autonomic failure is present in both motor subtypes correlating with neuronal degeneration in autonomic brainstem centres, intermediolateral cell columns and Onuf’s nucleus in the spinal cord [19,101]. The main pathological and diagnostic hallmark of MSA are AS-positive GCIs present in oligodendroglial cells and involving different areas of the brain, including pons, medulla, putamen, SN, cerebellum and preganglionic autonomic structures [3,102–105]. Moreover, astrogliosis and microgliosis were found to be involved in this presumably primary oligodendrogliopathy [19,27,106].

Similar to PD and DLB, the role of astroglial and microglial activation in MSA is not entirely resolved, taking neuroprotective and neurotoxic functions into account. Extensive astrogliosis in MSA brains was reported by Ozawa and colleagues [19] and by Jellinger et al. [107]. Astroglia in MSA brains undergo pathological changes regarding their morphology featuring enlarged cell bodies and distorted processes [108]. However, in contrast to PD and DLB, AS accumulation in astroglia does not seem to occur in MSA. Experimental MSA models provide further evidence on the involvement of astrogliosis in MSA-like neurodegeneration. Overexpression of human AS in oligodendroglia under the control of the murine myelin basic protein (MBP) promoter triggered neurodegeneration and prominent astrogliosis detected at 6 months of age [109]. Exposure to 3-nitropropionic acid in a transgenic mouse model overexpressing human AS in oligodendroglial cells under the control of the proteolipid protein (PLP) promoter not only lead to striatonigral degeneration and olivopontocerebellar atrophy but also to widespread astrogliosis accompanying the neurodegeneration [110]. Most of the in vitro data on astroglia and AS referenced for PD/DLB, as described above, may be also relevant for MSA. In MSA, astrocytes show high reactivity, and astrogliosis may lead to oxidative stress and neurotoxicity, similar to PD/DLB. However, insufficient data on astroglial activation and its mechanisms in MSA allow only speculations on the role of astroglial cells. Further studies would be very valuable for a complete understanding of the astroglial role in MSA.

Microglial activation in MSA is a common finding. In MSA patients, Gerhard and colleagues reported microglial activation in the dorsolateral prefrontal cortex, putamen, pallidum, pons and SN using [^11^C](R)-PK11195 PET imaging [27]. Moreover, an enhanced microgliosis was found in motor-related brain structures associated with GCI pathology, including cerebellar input, extrapyramidal motor and pyramidal motor structures [111]. Microglial activation was reproduced in the transgenic MSA mouse model overexpressing AS under a PLP promoter in oligodendroglial cells [110]. The progressive upregulation of microglial activation in this MSA transgenic mouse model resulted in neuroinflammation and oxidative stress correlating with dopaminergic neuronal loss in SNpc [112]. Moreover, the microglial activation was associated with an upregulation of TLR4 in these mice, as also detected in human MSA [112]. In double transgenic mice with oligodendroglial overexpression of AS and lack of functional TLR4, the efficiency of microglial AS clearance was diminished and resulted in enhanced nigral dopaminergic neurodegeneration [89]. These data suggest that TLR4 is a crucial mediator of microglial AS clearance, and the enhanced expression of this receptor in post-mortem brains may represent an augmented effort of AS clearance by microglial cells in MSA. In vitro data confirm that AS activates microglial cells, triggers the release of pro-inflammatory agents [76,82], increases oxidative stress through the release of ROS [84] and may be of equal relevance to the disease progression in PD and DLB as well as MSA. In summary, similar to PD and DLB, microglial cells in MSA display positive (phagocytosis) and negative (oxidative stress and inflammation) features, and further studies are warranted to elucidate the complete spectrum of microglial activation in disease initiation and progression.

It is considered that oligodendroglial cells play a leading role in MSA, due to the AS inclusions present in these cells. Oligodendrocytes seem to be initiators of the disease as regarded to the distribution of GCIs [106,113], which may even represent the primary injury in MSA [106,114]. GCIs are distributed throughout large proportions of the CNS [3,102–104]. However, the source of AS accumulation, the main component of GCIs, in oligodendroglial cells is not resolved yet. The prevalent assumption is that oligodendroglial cells actively incorporate and accumulate AS released by neighbouring neurons [115]. This hypothesis becomes highly relevant regarding the data on cell-to-cell propagation of AS in different studies [21–23]. Furthermore, the release of AS by neuronal cells into the extracellular space was confirmed recently [22,116]. Primary oligodendroglial dysfunction related to abnormal endocytic activity as suggested by the ectopic expression of Rab5 and Rabaptin-5 in GCIs [117] may represent an early event in MSA pathogenesis preceding pathological uptake and accumulation of AS in oligodendroglia. However, there are currently no studies demonstrating AS propagation to oligodendroglia [22,118]. Another possibility of AS aggregation in oligodendroglia could be an enhanced expression of AS, and further, a defective degradation mechanism could lead to accumulation of AS in the cell [119,120]. Yet, no mRNA expression of AS could be found in human oligodendroglial cells of MSA brains [113,121]. However, AS is a major trigger of oligodendroglial protein inclusion formation, and the absence of AS prevents accumulation of tau and αB-crystallin, further components of GCIs [122]. The oligodendroglial phosphoprotein p25α (tubulin polymerization promoting protein) induces AS aggregation in vitro [123]; furthermore, in MSA, p25α may relocate to oligodendroglial soma, suggesting an involvement of early oligodendroglial dysfunction in MSA initiation and GCI formation [124]. In support of these data, co-expression of human AS and p25α in rat oligodendroglia led to disorganisation of the microtubular cytoskeleton and apoptosis [125]. Inhibition of AS-Ser129 phosphorylation abolished these effects, suggesting an important role for Ser129 phosphorylation in the formation of AS oligomers and oligodendroglial apoptosis [125]. Recently, the cytoplasmic enzyme histone deacetylase 6 (HDAC6) was found in over 98 % of all GCIs of MSA post-mortem brains [126]. The exact role of HDAC6 in the pathogenic cascade of MSA is currently unclear. HDAC6 is identified to regulate the transport of ubiquitinated misfolded proteins, the formation of aggresomes [127] and aggresome degradation [128] as well as the control of autophagy pathways [129], and its accumulation in MSA may represent another sign of oligodendroglial injury.

Different studies demonstrate that GCIs affect oligodendroglial function and viability, suggesting an important role in MSA progression. Cell culture experiments, using glial cells overexpressing AS, revealed increased susceptibility to oxidative stress and TNF-α which may represent further events in the pathogenesis of MSA [130,131]. Overexpression of AS in oligodendroglial cells reduced the adhesion to fibronectin, leading to impaired cell–extracellular matrix interactions [132]. Oligodendroglial overexpression of AS in transgenic mice resulted in neurodegeneration in different brain areas including SNpc, locus coeruleus, nucleus ambiguous, pedunculopontine tegmental nucleus, laterodorsal tegmental nucleus and Onuf’s nucleus [110,133]. Moreover, MSA transgenic mouse models demonstrated that oligodendroglial AS inclusions may cause myelin disruption and mitochondrial dysfunction [109,112,134]. The expression of neurotrophic factors, especially GDNF, was decreased in the MBP-AS mouse model, but not in transgenic mice with neuronal overexpression of AS, suggesting MSA-specific oligodendroglial dysfunction, related to reduced trophic support of neurons [135].

In conclusion, oligodendroglial cells may play a major role in the initiation and progression of MSA (see Fig. 1). The accumulation of AS in these cells leads to altered oligodendroglial function including reduced trophic support and demyelination and in consequence to neurodegeneration. However, the source of AS in GCIs and the mechanisms of GCI formation in oligodendroglia remain unclear and need further elucidation to gain a deeper insight into MSA disease mechanisms.

## Therapeutic Approaches Targeting Glial Dysfunction in α-Synucleinopathies: Where Are We Now?

Increasing body of evidence confirms the relevance of glial dysfunction in the pathogenesis of α-synucleinopathies. However, the broad spectrum of activation profiles of microglial and astroglial cells makes it still difficult to obtain a clear-cut overall picture of all glial features and their mode of action in these diseases. The wide variety of glial functions offers diverse therapeutic targets. Yet, due to the insufficient knowledge on the exact chronology and relevance of the beneficial and detrimental roles of glia in α-synucleinopathies, researchers are currently confronted with discrepancies between findings on neuroprotection in experimental setups and clinical settings.

Neuroinflammatory responses and oxidative stress mediated by microglial or astroglial cells are prior targets in therapeutic approaches regarding neuroprotection in α-synucleinopathies. Non-steroidal anti-inflammatory drugs (NSAIDs) showed neuroprotective effects in toxin-induced PD-like neurodegeneration in rodents [136,137] and, furthermore, resulted in a decreased AS aggregation in vitro [138]. Recently, eicosanyl-5-hydroxytryptamide treatment lead to a repressed astro- and microglial activation and inducible nitric oxide synthase (iNOS) expression in AS-overexpressing mice [139]. Moreover, different strategies on the inhibition of micro- and astroglial activation were followed in various experimental approaches leading to neuroprotection, including iNOS and NADPH oxidase inhibition [140,141], suppression of the peroxisome proliferator-activated receptor γ pathway via pioglitazone [142–144] or inhibition of the enzyme MPO, which is involved in ROS production and upregulated in PD and MSA [61,145]. Minocycline, a tetracycline derivative known for its antimicrobial activity and the inhibition of protein synthesis, revealed additional anti-neuroinflammatory and anti-apoptotic efficacy [146]. In different experimental studies of PD and MSA, minocycline had various neuroprotective effects probably dependent on the timing of therapy onset within the course of neurodegeneration [112,147–149]. The modulation of TLR4-dependent microglial activation through a TLR4 antagonist naloxone has been suggested to prevent microgliosis-associated dopaminergic neurodegeneration [150,151]. However, in light of the recent finding that TLR4 is also an important modulator of AS clearance by microglia [89], caution and further studies are needed to justify such a therapeutic approach in α-synucleinopathies. Anti-neuroinflammatory strategies with the goal of modifying glial responses towards neuroprotection currently fail to translate into successful clinical trials. The application of NSAIDs in PD resulted in contradictory outcomes. In an epidemiological study, NSAID treatment was associated with decreased risk of PD [152]; however, in a recent study using a UK cohort of PD cases and controls [153] and in observational studies using meta-analysis [154,155], these results were not confirmed. A similar conflicting outcome was obtained after minocycline therapy in PD and MSA patients [156,157]. Minocycline treatment of MSA patients in a prospective, randomised, double-blind clinical trial lead to a significant downregulation of microglial activation after 24 weeks of therapy; however, no effect on disease progression was demonstrated, suggesting that an early therapy onset may be preferable [156].

Alternative therapeutic strategies, like immunomodulation, AS-reducing strategies and neurotrophic factor delivery and modulation, targeting glial dysfunction have been approached. In an experimental immunisation study, the adoptive transfer of copolymer-1 immune cells resulted in decreased microglial activation and enhanced local expression of astroglia-associated GDNF amongst other effects [158,159]. However, transfer of T cells from nitrated AS-immunised mice lead to neuroinflammation in correlation with neuronal loss [160]. In contrast, AS vaccination in a PD mouse model yielded a decrease in microglial and astroglial activation and enhanced neuroprotection, as well as reduced AS inclusion pathology [161]. The strategy of using AS-reducing agents for the therapy of α-synucleinopathies was further expanded by the application of rifampicin in a transgenic mouse model of MSA. The results indicated that the successful lowering of AS levels in the brain of MBP-AS mice resulted in neuroprotection associated with suppressed astroglial activation [162]. Clinical proof of concept is currently awaited for the efficacy of these strategies. Finally, the delivery of neurotrophic factors is a relevant approach related to glial dysfunction in α-synucleinopathies. Genetically modified macrophages were used for the delivery of GDNF inducing neuroprotection in the MPTP model of PD [163]. However, AAV gene delivery of a GDNF analogue in the putamen of PD patients failed to exert beneficial effects [164].

In conclusion, the divergence between the clinical and the experimental outcomes on therapies targeting glial dysfunction in α-synucleinopathies may be resolved only by further in-depth studies on the role of glial cells in disease initiation and progression. The role of glia should be further analysed in association with the basic changes that occur in CNS related to normal ageing, which may play a crucial predisposing/promoting role in AS-related neurodegeneration.
